# Cephalometric norms for the Saudi children living in the western region of Saudi Arabia: a research report

**DOI:** 10.1186/1746-160X-1-5

**Published:** 2005-08-24

**Authors:** Ali H Hassan

**Affiliations:** 1 P.O. Box 80209, Jeddah 21589, Preventive Dental Sciences Department, Faculty of Dentistry, King Abdulaziz University, Jeddah, Saudi Arabia

## Abstract

**Background:**

Previous studies have established specific cephalometric norms for children with different ethnic backgrounds, showing different facial features for each group. Up till now, there is a paucity of information about the cephalometric features of Saudi children living in the western region of Saudi Arabia, who have distinct social and climatic characteristics. The aim of the present study was to establish cephalometric norms for children living in the western region of Saudi Arabia.

**Methods:**

A total of 62 lateral cephalometric radiographs of Saudis (33 females and 29 males; aged 9–12 years) having good facial proportions and Class I dental occlusion, were traced and analyzed. Using the t-test, the mean value, standard deviation and the range of 20 angular and linear variables were calculated and compared to norms of adult Saudis living in the Western region of Saudi Arabia using the t-test. Male and female groups were also compared using the t- test.

**Results:**

Saudi children tend to have a significantly shorter and lower face height, a larger angle of convexity, and more proclined and protruded incisors when compared with adult Saudis (P < 0.05). There were no statistically significant differences between male and female groups.

**Conclusion:**

Saudi children have distinct cephalometric features, which should be used as a reference in the orthodontic treatment of young Saudi patients.

## Background

In orthodontic diagnosis and treatment planning, a cephalometric radiograph is an essential tool to relate patients with different malocclusions to their associated norms. Previous studies have established cephalometric norms for children in different countries who are descendants of special racial backgrounds [1-9]. Saudis were found to have distinct craniofacial features as compared with European-Americans [6-9]. Unfortunately, all the previously mentioned studies were performed in the central region of Saudi Arabia and there was only one study conducted in the western region, in which cephalometric norms were established for Saudi adults and then represented graphically on a wiggle to count for the variability of the readings among the Saudi population [9]. Results showed that Saudis, in general, have an increased ANB angle and bimaxillary protrusion when compared with European-American norms. It was concluded that the established norms should be used as a reference in the orthodontic treatment of Saudi adults. In addition, cephalometric norms should be presented on a polygon to count for the high variability that was observed in the Saudis because of their multiethnicity. A wiggle, as described by Vorhies and Adams [10], is a graph in which all average norms are plotted on a central vertical line. The maximum and the minimum readings of each norm are plotted on either side of the central line in a way that all the Class II readings are placed on the left side and the Class III readings are placed on the right side of the central line [10]. Unlike Vorhies and Admas [5], Hassan [9] used one standard deviation instead of the maximum and minimum readings of each reading.

The objectives of the present study were to establish norms for Saudi children living in the western region of Saudi Arabia and to present them graphically in the form of a polygon to count for any possible variation due to age, gender and multiracial background of the representing sample.

## Methods

The present study was approved by the Ethical Committee of the Faculty of Dentistry, King Abdulaziz University (KAAU), in which a total of 62 lateral cephalometric radiographs of Saudi children (33 females and 29 males; aged 9–12 years) having acceptable profiles with competent lips, Class I dental and skeletal relationships, minimum overbite and overjet, minimum or no crowding, and no previous orthodontic treatment were selected to be included in the study group. The selected subjects were Saudis (by nationality) born and living in the western region of Saudi Arabia and of Arab descent. They were selected through the public health program that was conducted by the Department of Preventive Dental Sciences at KAAU, in which primary and intermediate public schools were visited for caries assessment and patient oral health education.

**Figure 1:**
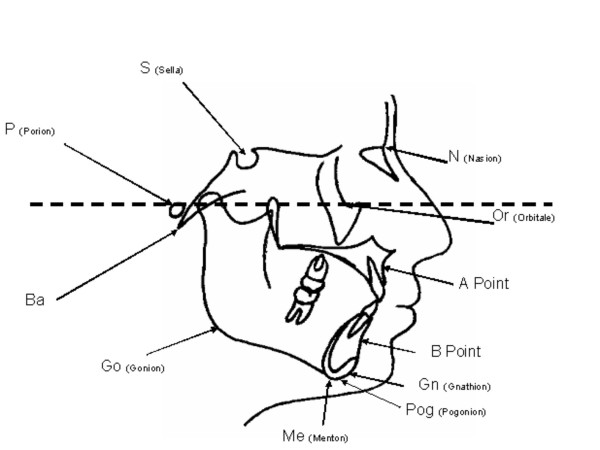
**Cephalometric reference points**. Different reference points used in the present study and their abbreviations.

**Figure 2:**
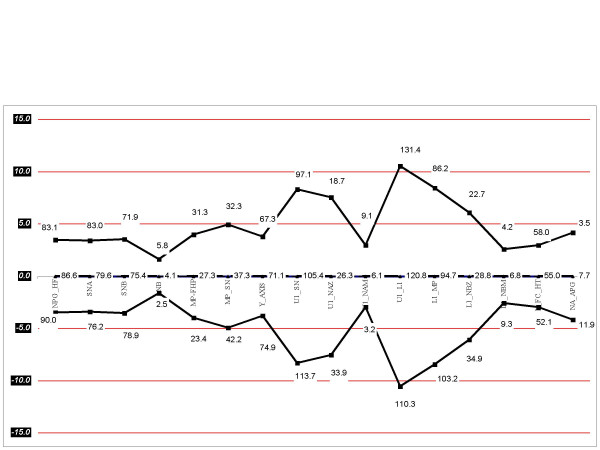
**Graphical presentation of cephalometric norms for Saudi children**. A graph (Wiggle) in which cephalometric norms for Saudi children (mean age: 12.2 years) are plotted on a central vertical line. The readings of plus or minus one standard deviation of each norm are plotted on either side of the central line in a way that all the Class II readings were placed on the left side and the Class III readings were placed on the right side of the central line.

The radiographs were traced and analyzed manually by a single examiner. Twenty angular and linear measurements were calculated (Table 1 and figure. 1). The mean value, standard deviation and range of each variable was calculated and compared with the norms established for Saudi adults living in the western region of Saudi Arabia [4]. In addition, measurements were compared between male and female children. An independent sample t-test was used in the comparison between children and adults and as well as between male and female groups. To assess tracing errors, a second tracing was prepared for every 10 tracings. The mean error in linear measurements was ± 0.35 mm. The mean error in angular measurements was ± 0.92°. A set of cephalometric values was established for Saudi children. The resulting data (means and standard deviation) were represented diagrammatically in the form of a polygon (Wiggle) (Figure 2) using the mean value plus or minus one standard deviation.

**Table 1:** Different linear and angular measurements used

NPog-FH	Intersection between NPog plane and Frankfort horizontal plane
NPog-FH	Intersection between NPog plane and Frankfort plane
SNA	Maxillary apical base relationship to anterior cranial base
SNB	Mandibular apical base relationship to anterior cranial base
ANB	Apical base relationship
NA-APog	Angle of convexity
	
MP-FH	Inclination of mandibular plane to FH
MP-SN	Inclination of mandibular plane angle to anterior cranial base
OC-PL-SN	Inclination of occlusal plane to anterior cranial base
Y-axis	Angle made between SN and NGn line
L-FC. Ht	Lower face height (Anterior nasal spine-Menton)
	
U1-SN	Inclination of maxillary incisors to anterior cranial base
U1-NAz	Inclination of maxillary incisors to NA
U1-NAmm	Protrusion of maxillary incisors to NA
U1-L1	Inclination of maxillary incisors to mandibular incisors
	
L1-MP	Inclination of mandibular incisors to mandibular plane
L1-NBz	Inclination of mandibular incisors to NB
L1-NBmm	Protrusion of maxillary incisors to NB
L1-APogz	Inclination of mandibular incisors to APog plane
L1-APogmm	Protrusion of mandibular incisors to APog plane

## Results

Table 2 and figure 2 show the mean and standard deviation of the 20 angular and linear measurements selected, which represent the norms established in the present study. As compared with adult Saudis, children have a significantly increased angle of convexity which indicates more convex profiles in the Saudi children (P < 0.05). In addition, the lower face height was significantly shorter in the children's group (P < 0.05). The ANB angle was insignificantly increased in the children's group (P < 0.05). Dentally, upper and lower incisors were significantly more proclined and more protruded in the children's group (P < 0.05). The other readings were generally similar between Saudi children and adults. In addition there were no statistically significant differences between Saudi males and females (P < 0.05) (Table 3).

**Table 2:** Cephalometric Standards for Saudi children

**Cephalometric Variables**	**Saudis (Children) n = 62**		**Saudis (Adults) n = 68**		**t**	**p**
			
	**Mean**	**SD**	**Mean**	**SD**		
NPog_FH	86.6	3.2	86.9	3.64	.054	0.957
NPog- SN	76.4	3.7	78.7	4.61	2.887	0.005*
SNA	79.6	5.4	80.8	4.06	.816	0.416
SNB	75.5	3.6	77.5	4.48	1.984	0.050
ANB	4.1	1.7	3.3	1.52	1.717	0.089
NA-APog	7.7	4.5	5.01	3.05	4.238	0.000*
MP/FH	27.3	4	28.5	4.79	1.103	0.272
MP/SN	37.2	5	35.9	5.96	1.940	0.055
Occl. Pl -SN	20.4	4.8	18.7681	6.21	.257	0.79
Y_AXIS	71.1	3.6	69.6	4.19	.460	0.647
L-F.HT	54.7%	1.198	56.0%	2.7	2.503	0.014*
U1_SN	105.5	5.6	106.8	8.07	2.922	0.004*
U1_NAZ	26.3	8.3	27.3	7.55	2.718	0.008*
U1_NAMM	6	1.8	6.8	2.92	4.491	0.000*
U1_L1	120.8	7.4	120.6	11.97	.277	0.782
L1_MP	94.7	6	93.9	7.7	2.276	0.025*
L1_NBZ	28.8	5.8	29.3	6.89	1.219	0.225
L1_NBMM	6.8	2.3	7.5	2.63	2.975	0.004*
L1_APogZ	26.5	5	27.23	6.07	0.564	0.574
L1_APogMM	4.5	1.97	4.89	2.89	2.064	0.042*

* P < 0.05

**Table 3:** Comparison of cephalometric measurements of the Saudi male and female children using t-test

**Cephalometric Variable**	**Male (n = 29)**		**Female (n= 33)**		**P**
		
	**Mean**	**SD**	**Mean**	**SD**	
NPog_FH	87.9	3.3	86.1	2.96	.64
NPog- SN	75.2	4.4	76.9	3.2	.42
SNA	74	5.1	77.8	4.98	.06
SNB	74.75	4.3	76.2	3.1	.38
ANB	3.8	1.7	3.9	1.8	.105
NA-APog	7	4.8	7.3	4.5	.058
MP/FH	25.4	4.5	26	3.8	.12
MP/SN	38	5.7	35	4.4	.418
Occ.P -SN	21.2	5.9	20	4.14	.174
Y_AXIS	70.4	4.1	69.5	3.4	.224
U1_SN	104.4	5.96	105.3	5.4	.095
U1_NAZ	28.6	8.2	25.4	5.5	.155
U1_NAMM	5.96	1.6	5.9	1.9	.062
U1_L1	122.2	7.6	120.95	7.4	.074
L1_MP	95.97	5.9	98.4	6	.224
L1_NBZ	28.7	5.3	29.3	6	.072
L1_NBMM	7.6	2.3	6.8	2.4	.471
L1_APogZ	25.5	4.5	26.96	5.3	.188
L1_APogMM	4.875	1.86	4.3	2	.496
Pg to NBMM	0.5	1.6	1.5	1.6	1.26

* P < 0.05

## Discussion

Considering the ethnic background of patients in setting treatment objectives is an important requirement for successful orthodontic treatment. This can be achieved by establishing cephalometric and facial norms for the different racial groups. Unfortunately, there are no cephalometric norms for Saudi children living in the western region of Saudi Arabia. This study is considered as the first trial to establish norms for Saudi children living in that region.

The Saudi race in the western region of Saudi Arabia is unique in composition, which is multiracial in nature and has been established through interbreeding among the different communities who migrated there to be close to the Holy mosques of the Islamic world [9]. The sample used in the present study was carefully selected to include Arab-Saudis born and living in the western region of the Kingdom of Saudi Arabia. Hassan (2006) found that Saudi adults have an increased facial convexity, a more convex profile, a steeper mandibular plane, more protruded upper and lower incisors and shorter lower face height as compared with European-Americans [9].

Results of the present study have shown that Saudi children have statistically different skeletal and dental features than Saudi adults living in the western region of Saudi Arabia. These differences were noticed in the facial plane angle, angle of convexity, lower face height and the inclination of incisors. Most of the differences go with the general growth pattern of the human face, in which chins are more retrognathic and profiles are more convex during childhood and tend to straighten by age [11] (Table 2). In addition, lower face height tends to increase with age during the transition from childhood to adulthood which could be attributed to the cephalo-caudal gradient of growth of the facial bones [12]. Dentally incisors tend to protrude and procline with age in the Saudis, which could be attributed to environmental factors such as the clinically observed high incidence of mouth breathing and tongue thrusting habits among Saudi youngsters.

Although the ANB angle is insignificantly increased in children as compared with adults, it is still considered as an important result to emphasize, for proper orthodontic diagnosis. An ANB angle of four degrees and an angle of convexity of seven degrees should be considered normal in children. In addition, an ANB angle of two degrees, which is considered normal in adults, should be investigated more in children to exclude the tendency for Skeletal Class III relationship.

## Conclusion

Saudi children living in the western region, have distinct facial and skeletal features which are different than Saudi adults. Therefore distinction should be made between young and adult patients. This can be achieved by using specific cephalometric norms for each age group.

## Competing interests

The author(s) declare that they have no competing interests.
